# Association between Serum Testosterone Levels and Body Composition among Men 20–59 Years of Age

**DOI:** 10.1155/2021/7523996

**Published:** 2021-09-20

**Authors:** Jiajie Ye, Xiaojun Zhai, Jinxiao Yang, Zhongxin Zhu

**Affiliations:** ^1^Liaoning University of Traditional Chinese Medicine, Shenyang, Liaoning 110847, China; ^2^Department of Orthopedics, The Affiliated Jiangnan Hospital of Zhejiang Chinese Medical University, Hangzhou, Zhejiang 311200, China; ^3^Department of Urology, The Xiaoshan Affiliated Hospital of Wenzhou Medical University, Hangzhou, Zhejiang 311200, China; ^4^Department of Osteoporosis Care and Control, The Xiaoshan Affiliated Hospital of Wenzhou Medical University, Hangzhou, Zhejiang 311200, China; ^5^Clinical Research Center, The Xiaoshan Affiliated Hospital of Wenzhou Medical University, Hangzhou, Zhejiang 311200, China

## Abstract

**Introduction:**

Sex hormones play an important role in the development and maintenance of bone and muscle mass. However, studies regarding serum testosterone levels, osteoporosis, and sarcopenia in men are relatively sparse and have led to contradictory conclusions. Therefore, this study aimed to investigate the association between serum testosterone levels and body composition, including bone mineral density (BMD), appendicular lean mass index (ALMI), and appendicular fat mass index (AFMI), among men 20–59 years of age through a cross-sectional analysis of the National Health and Nutrition Examination Survey.

**Materials and Methods:**

Our analysis was based on the data for 3,875 men, 20–59 years of age. Weighted multiple regression analyses were used to estimate the independent association between serum testosterone levels and body composition. Weighted generalized additive models and smooth curve fittings were used to characterize the nonlinear associations between them.

**Results:**

The association between the serum testosterone level and lumbar BMD was positive in each multivariable linear regression model. In the model adjusted for age and race, the serum testosterone level was negatively associated with ALMI. However, in the models adjusted for body mass index, this association became positive. In addition, the association between the serum testosterone level and AFMI was negative in each multivariable linear regression model.

**Conclusion:**

Our study demonstrated a positive association of serum testosterone level with lumbar BMD and ALMI, and a negative association with AFMI, among men 20–59 years of age, suggesting that increasing testosterone levels may be beneficial to skeletal health in young and middle-aged men with low testosterone levels.

## 1. Introduction

With aging of the general population, the prevalence of sarcopenia and osteoporosis is likely to increase substantially over the coming decades [1]. Sarcopenia is defined as the loss of skeletal muscle mass, quality, and strength, whereas osteoporosis is characterized by low bone mass and deterioration of the microarchitecture of bone [2, 3]. Both conditions are associated with devastating consequences, such as an increased risk of frailty, fragility fracture, multimorbidity, and other adverse outcomes [4]. Therefore, understanding the risk factors for these two conditions is essential for their prevention, early diagnosis, and management.

Sex hormones play an important role in the development and maintenance of bone and muscle mass [5, 6]. As one of the representative sex hormones, testosterone has recently been shown to be involved in several metabolic functions in males [7]. Adult hypogonadism is a well-known risk factor in the development of secondary osteoporosis [8]. While menopause-related estrogen deficiency is a well-studied risk factor for osteoporosis in women, data regarding serum testosterone levels and osteoporosis in men are less well known, especially in younger men <60 years of age. Similarly, studies investigating the efficacy of testosterone on muscle mass and function have yielded contradictory conclusions based on the method of testing and study cohort [9]. What is known is that lean mass and bone mass reach their peak in young adulthood in men followed by a slow rate decrease from middle-age onward [10, 11]. Therefore, the balance between the gain and loss of lean mass and bone mass during these periods may play a crucial role in the onset of sarcopenia and osteoporosis at an older age. Accordingly, this study aimed to evaluate the association between the serum testosterone level and body composition, including lumbar bone mineral density (BMD), appendicular lean mass index (ALMI), and appendicular fat mass index (AFMI) in younger individuals rather than elderly men, through a cross-sectional analysis of data from the National Health and Nutrition Examination Survey (NHANES).

## 2. Materials and Methods

### 2.1. Study Population

NHANES surveys have been conducted every 2 years in a nationally representative sample of the noninstitutionalized US population since 1999. To serve as a population-level assessment, the NHANES uses a complex, stratified, multistage probability sampling design. Data used in our study were obtained from the three waves of the NHANES conducted between 2011 and 2016.

The population was limited to men, 20–59 years of age (*n* = 5,540). Individuals with missing serum testosterone level data (*n* = 556), lumbar BMD data (*n* = 573), appendicular lean or fat data (*n* = 418), body mass index (BMI), or height data (*n* = 11), as well as patients with cancer (*n* = 94), were excluded. We further excluded 13 participants who had ever been prescribed medicine for osteoporosis or had ever taken prednisone or cortisone daily. After screening, the data for 3,875 men were included in the final analysis. All protocols of the NHANES were approved by the National Center for Health Statistics (NCHS), and all participants provided written consent for the use of their data for research.

### 2.2. Study Variables

The exposure variable in this study was the serum testosterone level. Serum samples were firstly collected between 8.30 a.m. and 11.30 a.m. following an overnight fast. Based on the reference method of the National Institute for Standards and Technology, the concentration of serum testosterone was measured using the isotope dilution-liquid chromatography tandem mass spectrometry (ID-LC-MS/MS) method, which was optimized for higher sample throughput [12]. Liquid-liquid extraction of serum was employed to isolate the steroid. As an internal standard, stable isotope-labeled testosterone was used to correct for sample recovery during the sample preparation process.

The outcome variable was body composition, including lumbar BMD, ALMI, and AFMI. Body composition was measured by dual-energy X-ray absorptiometry whole-body scans using Hologic Discovery model A densitometers (Hologic, Inc., Bedford, MA, USA) and analyzed using APEX software (version 4.0; Hologic, Inc., Bedford, MA, USA). In this study, ALMI was calculated as the appendicular lean mass (kg) divided by height squared (m^2^) and AFMI as the appendicular fat mass (kg) divided by height squared (m^2^).

Demographic variables including age, race/ethnicity, education level, income-to-poverty ratio, smoking status, drinking behavior, status of moderate activities, and calcium supplement use during the 30-day period prior to the survey date were obtained from the self-reported questionnaire. Body mass index (BMI) data were collected in the mobile examination center. As part of the standard biochemistry profile, blood urea nitrogen, serum uric acid, total protein, serum phosphorus, and serum calcium are included. Moreover, the serum estradiol level was also included. Details of serum testosterone levels, body composition measurements, and other covariate acquisition processes are available at https://www.cdc.gov/nchs/nhanes/.

### 2.3. Statistical Analyses

All estimates were calculated accounting for the NHANES sample weights, following the guidelines edited by the NCHS [13]. Weighted multiple regression analyses were used to estimate the independent association between serum testosterone levels and body composition. Three models were used to provide statistical inference: model 1, adjusted for age and race; model 2, adjusted for age, race, and BMI; and model 3, adjusted for all covariates.

To address potential nonlinearities, weighted generalized additive models and smooth curve fitting were performed. Statistical analyses were performed using Empower software (https://www.empowerstats.com; X&Y solutions, Inc., Boston, MA) and R version 3.4.3 (https://www.R-project.org, The R Foundation), with a *P* value < 0.05 as statistically significant.

## 3. Results

A total of 3,875 participants (20–59 years of age) were included in our analysis, with the weighted characteristics of the participants subclassified based on serum testosterone levels quartiles (Q1: ≤300.0 ng/dL; Q2: >300.0, ≤398.8 ng/dL; Q3: >399.0, ≤521.0 ng/dL; and Q4: >521.0 ng/dL), as shown in Table 1. There were significant differences in baseline characteristics between participants in the different serum testosterone levels quartiles, with the exception of race, education level, moderate activities, and calcium supplement use. Compared with the Q1 group, participants in the other quartile groups were younger, had increased total protein levels and lumbar BMD, and had decreased BMI, serum uric acid levels, AFMI, and ALMI.

### 3.1. Association between Serum Testosterone Level and Lumbar BMD

The association between the serum testosterone level and lumbar BMD was positive for each multivariable linear regression model (Table 2). Moreover, the trend remained significant among the different serum testosterone level quartile groups (*P* for trend <0.001). In the subgroup analysis stratified by age and race, this positive association remained in the 20- to 39-year group (*β* = 0.07, 95% CI: 0.02 to 0.11), and the non-Hispanic black (*β* = 0.09, 95% CI: 0.01 to 0.17) group, but not in the 40- to 59-year group (*β* = 0.01, 95% CI: −0.04 to 0.05).

### 3.2. Association between Serum Testosterone Level and ALMI

In the model adjusted for age and race (Table 3), the serum testosterone level was negatively associated with ALMI (*β* = −0.0022, 95% CI: −0.0024 to −0.0019). However, in the models adjusted for BMI, this association became positive (model 2: *β* = 0.0005, 95% CI: 0.0004 to 0.0006; model 3: *β* = 0.0007, 95% CI: 0.0005 to 0.0008). The *P* for trend remained significant in each model. In the subgroup analysis stratified by age and race, this positive association remained in both the 20–39 years (*β* = 0.0007, 95% CI: 0.0005 to 0.0009) and 40–59 years (*β* = 0.0006, 95% CI: 0.0004 to 0.0009) groups and in the non-Hispanic white (*β* = 0.0009, 95% CI: 0.0007 to 0.0012) and non-Hispanic black (*β* = 0.0004, 95% CI: 0.0001 to 0.0007) groups in the fully adjusted model.

### 3.3. Association between Serum Testosterone Level and AFMI

The association between the serum testosterone level and AFMI was negative in each multivariable linear regression model (Table 4). The *P* for trend remained significant in each model. In the subgroup analysis stratified by age and race, this negative association remained in both the 20–39 years (*β* = −0.0004, 95% CI: −0.0006 to −0.0002) and 40–59 years (*β* = −0.0003, 95% CI: −0.0005 to −0.0001) groups and in the non-Hispanic white (*β* = −0.0006, 95% CI: −0.0008 to −0.0003) group in the fully adjusted model.

**Figure 1:**
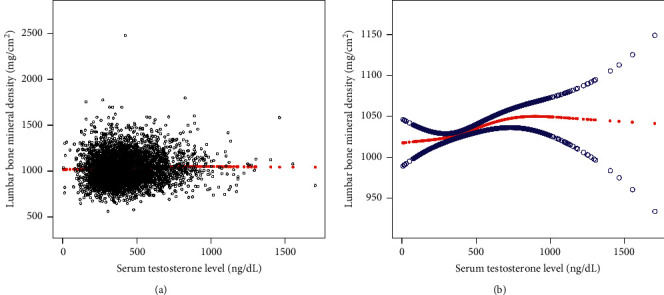
The association between serum testosterone levels and lumbar bone mineral density. (a) Each black point represents a sample. (b) Solid red line represents the smooth curve fit between variables. Blue bands represent the 95% confidence interval from the fit. Age, race, body mass index, education level, income-to-poverty ratio, smoking status, drinking behavior, moderate activities, calcium supplement use, blood urea nitrogen, serum uric acid, total protein, serum estradiol level, serum phosphorus, and serum calcium were adjusted.

**Figure 2:**
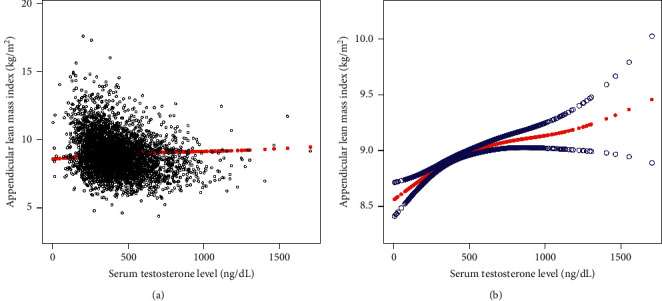
The association between serum testosterone levels and appendicular lean mass index. (a) Each black point represents a sample. (b) Solid red line represents the smooth curve fit between variables. Blue bands represent the 95% confidence interval from the fit. Age, race, body mass index, education level, income-to-poverty ratio, smoking status, drinking behavior, moderate activities, calcium supplement use, blood urea nitrogen, serum uric acid, total protein, serum estradiol level, serum phosphorus, and serum calcium were adjusted.

**Figure 3:**
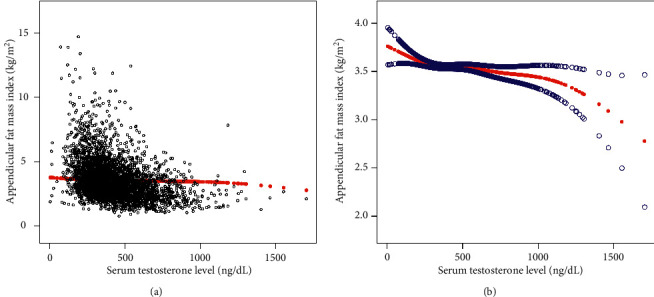
The association between serum testosterone levels and appendicular fat mass index. (a) Each black point represents a sample. (b) Solid red line represents the smooth curve fit between variables. Blue bands represent the 95% confidence interval from the fit. Age, race, body mass index, education level, income-to-poverty ratio, smoking status, drinking behavior, moderate activities, calcium supplement use, blood urea nitrogen, serum uric acid, total protein, serum estradiol level, serum phosphorus, and serum calcium were adjusted.

Results of the smooth curve fitting and generalized additive models to characterize the nonlinear associations between serum testosterone levels and lumbar BMD, ALMI, and AFMI are shown in Figures 1–3.

## 4. Discussion

Our population-based study revealed an association between serum testosterone levels and multiple indicators of body composition in men 20–59 years of age, namely a positive association with lumbar BMD and ALMI and a negative association with AFMI.

Osteoporosis in men is a growing public health concern, which remains underdiagnosed and underappreciated [14]. Sex hormones play an important role not only in the acquisition of bone mass but also in the maintenance of bone mass [15]. In a study of 60 schoolboys, the serum testosterone level was a major determinant of BMD at different pubertal stages [16]. In another study, a positive association between serum testosterone levels at the age of 12 years and a subsequent 6-year increase in BMD was identified [17]. A recent randomized clinical trial of men ≥65 years of age reported that increasing the testosterone concentration among older men with low testosterone levels has a positive effect on BMD and estimated bone strength [18]. One study specifically among men <50 years of age revealed that men with testosterone deficiency had greater odds of osteopenia and osteoporosis, with increases in BMD with testosterone treatment [19]. However, a recent meta-analysis showed no significant difference in total testosterone levels between primary osteoporotic and nonosteoporotic males [20].

It is well known that bodybuilders and athletes abuse testosterone to develop extra muscle bulk and strength [21]. However, testosterone under normal physiological conditions also helps regulate muscle mass and strength [22]. One study investigating the effect of long-acting testosterone in patients with congestive cardiac failure reported an improvement in muscle strength [23]. The findings from a previous NHANES (1999–2000) study of men, aged 18–85 years, revealed that higher testosterone at physiologic levels was associated with higher body lean mass and lower body fat mass [24]. However, other studies did not identify an effect on muscle strength [25, 26]. In our study, the serum testosterone level was negatively associated with ALMI in the BMI-unadjusted model. However, in the models adjusted for BMI, this association became positive. There are a variety of techniques used to estimate muscle quantity or mass, and multiple methods of adjusting the results for height or BMI. Our data showed that adjusting for height or BMI has a fundamental impact on the results. Therefore, these inconsistent findings related to the association between serum testosterone levels and BMD, and ALMI, may be attributed to heterogeneity among studies, including differences in study designs, participant selection, and control of confounding factors, especially BMI.

On the other hand, testosterone has been shown to exert an inhibitory effect on the incorporation of dietary fat into adipose tissue [27]. Testosterone also contributes to the maintenance of lower levels of fat mass by its conversion to estradiol [28]. A recent study of hypogonadal men reported an impressive effect of testosterone therapy on body weight, waist circumference, and BMI when compared with those not treated with testosterone [29].

The exact mechanism underpinning the association between testosterone and bone metabolism remains unclear. One possible explanation is that testosterone modulates bone remodeling either via aromatization of testosterone to estradiol or through direct activating of sex steroid receptors in bone cells [30]. Moreover, testosterone may have an indirect effect on bone mass through its anabolic effect of increasing muscle mass [31]. For individuals with low testosterone levels, adequate testosterone supplementation may be a useful adjunct therapy; however, the safety of testosterone intervention is of paramount concern as testosterone is a potent hormone acting on various organ systems.

To the best of our knowledge, our study is the largest report on the association between serum testosterone level and body composition in younger adult men <60 years of age. Our use of a large national representative sample increases the statistical strength for increased reliability of our results. Moreover, our age-stratified subgroup analysis allowed us to observe the association between the serum testosterone level and body composition in different life stages of youth and middle age. However, the limitations of our study need to be acknowledged. First, the cross-sectional design limits the inference of a causal correlation between the serum testosterone level and body composition. Second, because serum testosterone was not a static but a dynamic variable, measuring testosterone only once may cause some bias in testosterone levels used in the analysis. Third, in men's circulating plasma testosterone, approximately 2% to 3% is in free form, 33% to 54% is bound to albumin, and 44% to 65% is bound to sex hormone binding globulin, which loses its biological activity because of tight binding [32]. Bioavailable testosterone levels are not completely associated with testosterone levels. In our study, the association among free testosterone, bioactive testosterone or free androgen index, and body composition was not analyzed; this should be further clarified in future research.

In conclusion, we report a positive association between the serum testosterone level and lumbar BMD and ALMI, with a negative association to AFMI among men 20–59 years of age. Therefore, increasing testosterone levels may be beneficial to skeletal health in young and middle-aged men who have low testosterone levels. Further studies are needed to explore the threshold testosterone level that is beneficial for skeletal health without causing adverse events.

## Figures and Tables

**Table 1:** Weighted characteristics of study population based on serum testosterone levels quartiles.

Serum testosterone levels (ng/dL)	Q1 (≤300.0)	Q2 (>300, ≤398.8)	Q3 (>399.0, ≤521)	Q4 (>521.0)	*P* value
Age (years)	40.8 ± 10.8	39.6 ± 11.5	38.2 ± 11.7	36.2 ± 11.9	<0.001
Race/ethnicity (%)					0.2914
Non-Hispanic white	62.6	60.5	63.2	60.0	
Non-Hispanic black	9.2	10.2	9.5	13.0	
Mexican American	11.6	12.6	11.2	11.1	
Other race/ethnicity	16.5	16.6	16.1	15.9	
Education level (%)					0.4021
Less than high school	14.8	14.7	16.1	16.5	
High school	22.0	24.1	22.0	24.9	
More than high school	63.2	61.1	61.9	58.6	
Body mass index (kg/m^2^)	31.9 ± 6.7	29.2 ± 5.2	27.3 ± 4.6	25.7 ± 4.6	<0.001
Income-to-poverty ratio	3.1 ± 1.6	3.0 ± 1.6	2.9 ± 1.6	2.7 ± 1.6	<0.001
Moderate activities (%)					0.399
Both moderate recreational and work activities	21.6	22.2	22.9	24.2	
Moderate recreational activities	23.6	22.9	24.8	24.2	
Moderate work activities	25.6	26.0	21.3	23.0	
No moderate recreational or work activities	29.2	28.9	30.9	28.6	
Smoking status (%)					<0.001
Everyday	12.4	17.0	18.2	22.5	
Some day	4.9	5.6	6.0	9.1	
Past smoking	27.0	20.8	21.7	19.1	
Nonsmoker or smoked less than 100 cigarettes in life	55.7	56.6	54.0	49.3	
Had ≥12 alcohol drinks per year (%)					0.026
Yes	80.69	82.88	83.41	85.54	
No	15.51	12.24	12.60	10.10	
Not recorded	3.80	4.88	3.99	4.36	
Blood urea nitrogen (mmol/L)	4.82 ± 1.64	4.87 ± 1.48	4.80 ± 1.39	4.67 ± 1.48	0.029
Serum uric acid (*μ*mol/L)	378.3 ± 78.2	364.8 ± 69.0	355.9 ± 63.7	335.7 ± 64.6	<0.001
Total protein (g/L)	71.6 ± 4.5	71.7 ± 4.5	71.8 ± 4.3	72.3 ± 4.4	0.006
Serum estradiol level (pg/mL)	21.2 ± 8.4	22.4 ± 7.2	24.1 ± 7.3	29.7 ± 10.1	<0.001
Serum phosphorus (mmol/L)	1.21 ± 0.18	1.21 ± 0.18	1.19 ± 0.18	1.19 ± 0.19	0.042
Serum calcium (mmol/L)	2.36 ± 0.09	2.36 ± 0.08	2.36 ± 0.08	2.37 ± 0.08	<0.001
Calcium supplement use 30 days (%)					0.2892
≤400 (mg)	24.6	25.0	24.8	23.1	
>400 (mg)	12.1	10.3	10.0	9.0	
Not recorded	63.3	64.7	65.2	67.9	
Appendicular fat mass index (kg/m^2^)	4.4 ± 1.8	3.7 ± 1.2	3.3 ± 1.2	2.9 ± 1.1	<0.001
Appendicular lean mass index (kg/m^2^)	9.4 ± 1.5	9.0 ± 1.3	8.7 ± 1.2	8.5 ± 1.3	<0.001
Lumbar bone mineral density (mg/cm^2^)	1017.3 ± 154.6	1017.5 ± 153.4	1035.3 ± 149.3	1047.6 ± 149.5	<0.001

Mean ± SD for continuous variables: *P* value was calculated by the weighted linear regression model. % for categorical variables: *P* value was calculated by the weighted chi-square test.

**Table 2:** Association between serum testosterone levels (ng/dL) and lumbar bone mineral density (mg/cm^2^).

	Model 1 *β* (95% CI)	Model 2 *β* (95% CI)	Model 3 *β* (95% CI)
Serum testosterone levels (ng/dL)	0.04 (0.02, 0.07)^*∗∗*^	0.07 (0.04, 0.10)^*∗∗∗*^	0.04 (0.01, 0.07)^*∗∗*^

*Serum testosterone levels (quartile)*			
Q1 (≤300.0)	Reference	Reference	Reference
Q2 (>300, ≤398.8)	−0.93 (−14.11, 12.25)	3.83 (−9.54, 17.20)	0.20 (−13.24, 13.63)
Q3 (>399.0, ≤521)	16.20 (3.03, 29.37)	24.14 (10.39, 37.88)	19.28 (5.30, 33.26)
Q4 (>521.0)	24.27 (10.93, 37.62)	34.84 (20.49, 49.18)	23.59 (8.10, 39.08)
*P* for trend	<0.001	<0.001	<0.001

*Stratified by age*			
20–39 years	0.06 (0.03, 0.10)^*∗∗*^	0.08 (0.05, 0.12)^*∗∗∗*^	0.07 (0.02, 0.11)^*∗∗*^
40–59 years	0.02 (−0.03, 0.06)	0.04 (−0.00, 0.09)	0.01 (−0.04, 0.05)

*Stratified by race*			
Non-Hispanic white	0.05 (0.00, 0.09)^*∗*^	0.06 (0.01, 0.11)^*∗*^	0.04 (−0.02, 0.09)
Non-Hispanic black	0.05 (−0.02, 0.11)	0.09 (0.02, 0.16)^*∗∗*^	0.09 (0.01, 0.17)^*∗*^
Mexican American	0.04 (−0.02, 0.10)	0.05 (−0.02, 0.11)	0.02 (−0.05, 0.09)
Other race/ethnicity	0.04 (−0.01, 0.09)	0.07 (0.02, 0.12)^*∗*^	0.03 (−0.03, 0.09)

Model 1: age and race were adjusted. Model 2: age, race, and body mass index were adjusted. Model 3: age, race, body mass index, education level, income-to-poverty ratio, smoking status, drinking behavior, moderate activities, calcium supplement use, blood urea nitrogen, serum uric acid, total protein, serum estradiol level, serum phosphorus, and serum calcium were adjusted. In the subgroup analysis stratified by race, the model is not adjusted for the stratification variable itself. ^*∗*^*P* < 0.05, ^*∗∗*^*P* < 0.01, and ^*∗∗∗*^*P* < 0.001.

**Table 3:** Association between serum testosterone levels (ng/dL) and appendicular lean mass index (kg/m^2^).

	Model 1 *β* (95% CI)	Model 2 *β* (95% CI)	Model 3 *β* (95% CI)
Serum testosterone levels (ng/dL)	−0.0022 (−0.0024, −0.0019)^*∗∗∗*^	0.0005 (0.0004, 0.0006)^*∗∗∗*^	0.0007 (0.0005, 0.0008)^*∗∗∗*^

*Serum testosterone levels (quartile)*			
Q1 (≤300.0)	Reference	Reference	Reference
Q2 (>300, ≤398.8)	−0.4736 (−0.5890, −0.3583)	0.0817 (0.0168, 0.1466)	0.0891 (0.0252, 0.1530)
Q3 (>399.0, ≤520.5)	−0.7957 (−0.9110, −0.6805)	0.1310 (0.0644, 0.1977)	0.1628 (0.0963, 0.2293)
Q4 (≥521.0)	−0.9918 (−1.1086, −0.8750)	0.2418 (0.1722, 0.3115)	0.3006 (0.2270, 0.3743)
*P* for trend	<0.001	<0.001	<0.001

*Stratified by age*			
20–39 years	−0.0022 (−0.0026, −0.0019)	0.0006 (0.0004, 0.0008)^*∗∗∗*^	0.0007 (0.0005, 0.0009)^*∗∗∗*^
40–59 years	−0.0019 (−0.0023, −0.0016)	0.0004 (0.0002, 0.0006)^*∗∗∗*^	0.0006 (0.0004, 0.0009)^*∗∗∗*^

*Stratified by race*			
Non-Hispanic white	−0.0020 (−0.0023, −0.0016)^*∗∗∗*^	0.0007 (0.0004, 0.0009)^*∗∗∗*^	0.0009 (0.0007, 0.0012)^*∗∗∗*^
Non-Hispanic black	−0.0027 (−0.0032, −0.0022)^*∗∗∗*^	0.0005 (0.0002, 0.0007)^*∗∗*^	0.0004 (0.0001, 0.0007)^*∗*^
Mexican American	−0.0023 (−0.0028, −0.0017)^*∗∗∗*^	0.0002 (−0.0001, 0.0005)	0.0003 (−0.0000, 0.0007)
Other race/ethnicity	−0.0023 (−0.0027, −0.0018)^*∗∗∗*^	0.0001 (−0.0001, 0.0004)	0.0001 (−0.0002, 0.0003)

Model 1: age and race were adjusted. Model 2: age, race, and body mass index were adjusted. Model 3: age, race, body mass index, education level, income-to-poverty ratio, smoking status, drinking behavior, moderate activities, calcium supplement use, blood urea nitrogen, serum uric acid, total protein, serum estradiol level, serum phosphorus, and serum calcium were adjusted. In the subgroup analysis stratified by race, the model is not adjusted for the stratification variable itself. ^*∗*^*P* < 0.05, ^*∗∗*^*P* < 0.01, and ^*∗∗∗*^*P* < 0.001.

**Table 4:** Association between serum testosterone levels (ng/dL) and appendicular fat mass index (kg/m^2^).

	Model 1 *β* (95% CI)	Model 2 *β* (95% CI)	Model 3 *β* (95% CI)
Serum testosterone levels (ng/dL)	−0.0032 (−0.0034, −0.0029)^*∗∗∗*^	−0.0003 (−0.0004, −0.0001)^*∗∗∗*^	−0.0003 (−0.0005, −0.0002)^*∗∗∗*^

*Serum testosterone levels (quartile*)			
Q1 (≤300.0)	Reference	Reference	Reference
Q2 (>300, ≤398.8)	−0.6786 (−0.7988, −0.5584)	−0.0714 (−0.1308, −0.0120)	−0.0745 (−0.1337, −0.0153)
Q3 (>399.0, ≤520.5)	−1.0777 (−1.1978, −0.9576)	−0.0643 (−0.1253, −0.0033)	−0.0775 (−0.1391, −0.0158)
Q4 (≥521.0)	−1.4748 (−1.5965, −1.3531)	−0.1259 (−0.1896, −0.0622)	−0.1482 (−0.2165, −0.0799)
*P* for trend	<0.001	<0.001	0.001

*Stratified by age*			
20–39 years	−0.0036 (−0.0040, −0.0033)^*∗∗∗*^	−0.0003 (−0.0005, −0.0001)^*∗∗∗*^	−0.0004 (−0.0006, −0.0002)^*∗∗∗*^
40–59 years	−0.0026 (−0.0029, −0.0022)^*∗∗∗*^	−0.0002 (−0.0004, −0.0001)^*∗*^	−0.0003 (−0.0005, −0.0001)^*∗∗*^

*Stratified by race*			
Non-Hispanic white	−0.0033 (−0.0037, −0.0029)^*∗∗∗*^	−0.0004 (−0.0007, −0.0002)^*∗∗∗*^	−0.0006 (−0.0008, −0.0003)^*∗∗∗*^
Non-Hispanic black	−0.0036 (−0.0041, −0.0030)^*∗∗∗*^	−0.0003 (−0.0006, −0.0000)^*∗*^	−0.0002 (−0.0005, 0.0000)
Mexican American	−0.0029 (−0.0035, −0.0022)^*∗∗∗*^	0.0001 (−0.0003, 0.0004)	0.0001 (−0.0003, 0.0005)
Other race/ethnicity	−0.0024 (−0.0028, −0.0020)^*∗∗∗*^	0.0001 (−0.0001, 0.0003)	0.0002 (−0.0000, 0.0004)

Model 1: age and race were adjusted. Model 2: age, race, and body mass index were adjusted. Model 3: age, race, body mass index, education level, income-to-poverty ratio, smoking status, drinking behavior, moderate activities, calcium supplement use, blood urea nitrogen, serum uric acid, total protein, serum estradiol level, serum phosphorus, and serum calcium were adjusted. In the subgroup analysis stratified by race, the model is not adjusted for the stratification variable itself. ^*∗*^*P* < 0.05, ^*∗∗*^*P* < 0.01, and ^*∗∗∗*^*P* < 0.001.

## Data Availability

The data of this study are publicly available on the NHANES website.
